# Alleviating the NF-κB/NLRP3 pathway-mediated pyroptosis and ameliorating the cognitive function of aged mice post partial hepatectomy by increasing the Bmal1 level via subanesthetic doses of ketamine

**DOI:** 10.1515/tnsci-2025-0370

**Published:** 2025-08-20

**Authors:** Wenbin Zeng, Xiaoming Lei, Hongtao Liu, Simin Zheng, Qianru Wang, Xiaoli Niu

**Affiliations:** Department of Anesthesia, Shaanxi Provincial Cancer Hospital, Xi’an, 710061, Shaanxi, China; Department of Anesthesia, The Second Affiliated Hospital of Xi’an JiaoTong University, Xi’an, 710004, Shaanxi, China; Department of Anesthesia, The Second Affiliated Hospital of Xi’an JiaoTong University, 157 Xi Wu Road, Xi’an, 710004, Shaanxi, China

**Keywords:** subanesthetic doses, ketamine, Bmal1, NF-κB/NLRP3 pathway, cognitive functions

## Abstract

**Background:**

As a non-competitive blocker of the *N*-methyl-d-aspartate receptor, ketamine is widely used for anesthesia and pain relief in clinical settings. However, certain neurological side effects may appear if it is used for the long term. According to clinical observations, anesthetic doses of ketamine trigger postoperative neurocognitive dysfunction in elderly patients, while subanesthetic doses of ketamine suppress the postoperative neuronal pyroptosis in the hippocampus, ameliorating the cognitive function. There is a certain link between brain and muscle arnt-like 1 (Bmal1) and the postoperative cognitive functions of elderly patients. Meanwhile, the Bmal1 activity can be intensified by subanesthetic doses of ketamine. How subanesthetic doses of ketamine act on the postoperative cognitive functions of elderly patients via Bmal1 is to be further investigated.

**Methodology:**

To expound how different doses of ketamine affect the cognitive functions of 15-month-old mice (No.: C57BL/6) receiving partial hepatectomy (PH), the following assays were conducted: (1) Morris Water Maze tests were made on mice on days1, 3, and 7 post-surgery; (2) histopathological analyses (by Nissl and Tunel staining) as well as western blotting, immunofluorescence, immunohistochemical, and ELISA assays were carried out on the hippocampal tissue samples collected from the mice 3 days post-surgery. Furthermore, to verify the critical role of the Bmal1 gene in the subanesthetic doses of ketamine-based improvement of cognitive function in aged mice post-surgery, the survey on 15-month-old mice (No.: C57BL/6) with inactivated Bmal1 gene was continued. Through the aforementioned assays, the modulation mechanism of subanesthetic doses of ketamine in ameliorating postoperative cognitive functions of aged mice was elucidated.

**Results:**

As revealed through this investigation, subanesthetic doses of ketamine compared with the sham group effectively enhanced mice’s memory and learning ability, increased the expression of p-NR2B and BDNF proteins, mitigated neuronal pathologic injuries and neuroinflammation in aged mice post-surgery, upregulated the gene expression of Bmal1, and inhibited the hippocampal neuronal pyroptosis mediated by the NF-κB/NLRP3 signaling pathway. These effects are contrary to those of anesthetic doses of ketamine. Furthermore, for mice with an inactivated Bmal1 gene, injecting subanesthetic doses of ketamine helped to alleviate neuronal pathological injuries and neuroinflammation in the hippocampal tissues, suppress the expression of cytokines pertaining to the NF-κB/NLRP3 signaling pathway, and improve postoperative memory and learning competence of the mice.

**Conclusion:**

Subanesthetic doses of ketamine can elevate the expressed level of Bmal1, dampening the NF-κB/NLRP3 pathway-mediated cell pyroptosis, alleviating neuroinflammation, and improving the postoperative cognitive functions of aged mice. The findings in this study suggest a novel approach to explore using subanesthetic doses of ketamine to ameliorate the cognitive functions of aged patients post-surgery and clinically prevent postoperative neurocognitive disorders.

## Graphical abstract

**Figure:**
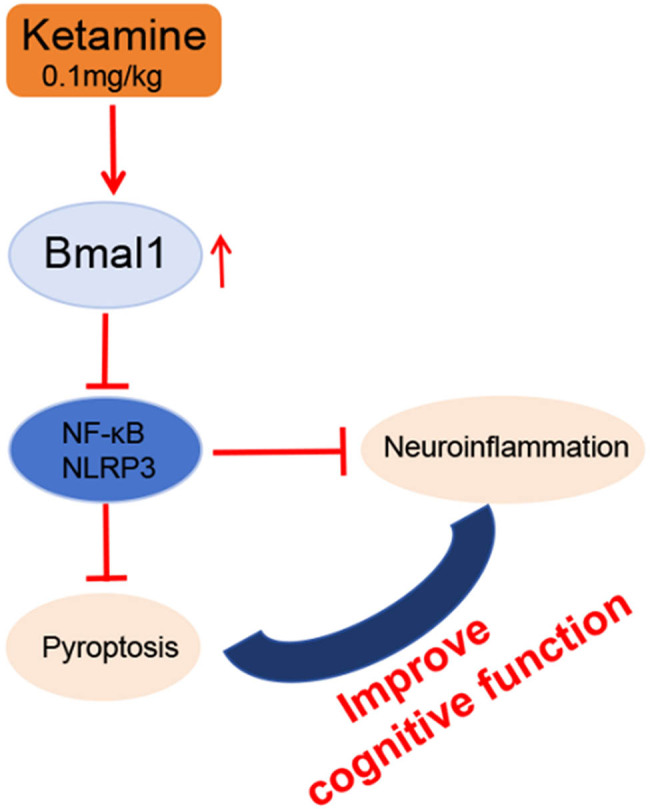

Subanesthetic dose of ketamine can alleviate pyroptosis and neuroinflammation mediated by the NF-κB/NLRP3 pathway via upregulating Bmal1 expression, thereby alleviating cognitive function after partial hepatectomy in elderly mice.

## 1 Introduction

A widespread neurological disorder, cognitive dysfunction, presenting clinically with decreased sensitivity and a downturn in learning and memory, has not been treated satisfactorily [1,2]. A critical pathological feature of this condition is the progressive loss and impaired function of hippocampal neurons, which is strongly tied to neuroinflammatory activities in the central nervous system (CNS) [3–5]. Elderly patients, particularly those above 60 years, are at a higher risk of suffering from postoperative neurocognitive disorder (PND) [6], where the severity of this disorder is evidently related to the patient’s age [7]. The pathogenesis of PND is not yet definite, but it is likely in close relation with inflammatory responses in the CNS. Ketamine, a common anesthetic drug used in clinical practice, may induce PND if administered at anesthetic doses [8,9], but can significantly alleviate this condition if administered at subanesthetic doses [10]. Given that ketamine is a non-selective *N*-methyl-d-aspartate (NMDA) receptor inhibitor [11], the effects mentioned are not likely due to differential actions on various subunits of the NMDA receptor. Thus, it was hypothesized that different doses of ketamine may modulate the expressed level of a certain gene, thereby affecting the gene’s downstream inflammatory signaling pathways and exerting divergent effects on the postoperative cognitive functions of aged mice.

Ketamine is a commonly used intravenous anesthetic in clinical practice, but it is prone to mental side effects such as nightmares and hallucinations. With the discovery of new indications and mechanisms of action, subanesthetic doses of ketamine can increase the level of dopamine in the body, thereby improving the postoperative cognitive function of patients. The more recognized subanesthetic dose of ketamine is defined as follows: single intravenous injection or epidural space <1 mg/kg; continuous intravenous injection <20 μg/kg·min; and single intramuscular injection <2 mg/kg. In clinical studies, low-dose ketamine can alleviate postoperative cognitive dysfunction after cardiac surgery by reducing inflammatory injury [10]. In a randomized controlled trial, low-dose ketamine can reduce postoperative neurocognitive dysfunction in elderly patients undergoing general anesthesia for gastrointestinal tumors to some extent [12]. Ketamine at 0.5 mg/kg alleviated oxidative stress, inflammatory response, and neuronal degeneration by activating the TRPV4/AMPK/NF-κB signaling pathway, thereby improving neurocognitive impairment in elderly mice after tibial fracture surgery [13]. Therefore, a subanesthetic dose of ketamine can be used to improve postoperative cognitive dysfunction in elderly patients.

Bmal1 (namely the brain and muscle arnt-like 1), a circadian clock gene, is the only gene that can cause complete loss of circadian rhythm in mice if this gene is inactivated [14,15]. The expressed level of the Bmal1 gene is negatively correlated with the patient’s age [16]. Knocking the Bmal1 gene out causes not only changes in the circadian rhythms but also pronounced premature aging symptoms, ultimately reducing the lifespan of mice; conversely, a higher level of Bmal1 can significantly alleviate these symptoms of early aging [17]. In the CNS of elderly individuals, the Bmal1 gene is less expressed, making them more susceptible to PND than the young. As pointed out in articles published recently, Bmal1 can negatively regulate the clock level, while the clock directly promotes NF-κB activation [18]; the expressed level of the Bmal1 gene is inversely related to that of NF-κB [19].

Based on the existing articles published, the NF-κB/NLRP3 signaling pathway functions crucially in the process of pyroptosis [20]. NF-κB represents the nuclear transcription factor кB and primarily acts in such processes as inflammation, cellular immunity, and pyroptosis [21]. If NF-κB is activated, the inactive NLRP3s may oligomerize, forming the NLRP3 inflammasome. NLRP3 represents the pyrin domain-containing protein 3 of the nucleotide-binding oligomerization domain-like receptor family. The NLRP3-mediated inflammatory responses are significant for neurological diseases [22–25]: elevating the level of NLRP3 can trigger the maturation and subsequent release of such inflammatory cytokines as IL-1β, promoting inflammatory responses [26], while restraining NLRP3 or components of the inflammasome helps to ease neuroinflammation and neurodegenerative changes, thereby greatly improving the cognitive functions [27–29]. Hence, diminishing the NF-κB/NLRP3 pathway and its downstream inflammatory factors is promising to be effective in treating CNS diseases. NLRP3 inflammasome is found to be highly expressed and excessively activated in aged mice [30]. In central inflammatory responses, the activated NLRP3 inflammasome functions vitally: it not only boosts the development of PND but also relates closely to infections and pains post-surgery and psychiatric disorders [31]. However, the association of pyroptosis with PND in elderly patients has not yet been probed into.

In this research, the influences of subanesthetic doses of ketamine on the postoperative memory and learning ability, hippocampal tissue pathological injuries, and neuronal pyroptosis in aged mice before and after knocking the Bmal1 gene out are determined. Aged mice were employed to model clinical scenarios in elderly patients to understand how subanesthetic doses of ketamine influence postoperative cognitive functions in aged mice by modulating the Bmal1 gene to interfere with neuronal pyroptosis. Further, this study delved into how the Bmal1 gene regulates the pyroptosis in the hippocampal neurons of aged mice via the NF-κB/NLRP3 signaling pathway.

## 2 Materials and methodology

### 2.1 Animals and grouping for testing

This survey drew on research methods reported in the literature [32]. The Laboratory Animal Center of the First Affiliated Hospital of PLA General Hospital (Beijing, China) provided 40 SPF-grade female mice numbered C57BL/6 (specifications: 15-month old; 25–35 g). These mice were randomly grouped into six groups (ten mice per group): a group receiving sham surgery (sham group), a group injected with normal saline (NS group), a group receiving injection with anesthetic doses of ketamine (anesthetic group), a group receiving injection with subanesthetic doses of ketamine (subanesthetic group), a group receiving injection with subanesthetic doses of ketamine + NF-κB agonists BAY 11-7085 (subanesthetic + BAY group), and a group receiving injection with subanesthetic doses of ketamine + NLRP3 agonist Nigericin (subanesthetic + Nig group). Except for the sham group, the remaining five model groups underwent partial hepatectomy (PH). One hour before surgery, the sham group and the NS group were injected with 1 mL of normal saline; the anesthetic and subanesthetic groups were administered intraperitoneally with 1 and 0.1 mg/kg of ketamine, respectively. One hour after operation, 5 μg/kg BAY 11-70851 and 1 mL/kg Nigericin were injected intraperitoneally in the subanesthetic + BAY group and subanesthetic + Nig group, respectively.

To assess the way Bmal1 affects aged mice’s cognitive functions, 30 female mice numbered C57BL/6 with identical identifications were selected from the Chinese Academy of Sciences (Shanghai, China) affiliated Laboratory Animal Center. For facilitating the tests, the 30 mice were classified equally into three groups: a group of wild-type mice with active Bmal1 gene (WT group), a group of mice with Bmal1 gene deactivated (Bmal1-KO group), and a group of mice with Bmal1 gene deactivated and receiving injection with subanesthetic doses of ketamine (Bmal1-KO + subanesthetic group). All three groups experienced PH. One hour prior to surgery, the WT and Bmal1-KO groups received an intraperitoneal injection of 1 mL normal saline; the Bmal1-KO + subanesthetic group received an intraperitoneal injection of 0.1 mg/kg ketamine. The mice were accommodated in a quiet environment with adequate ventilation and air filtration systems, at the following settings: 20–22°C (room temperature); around 50% relative humidity; and alternated 12-h light and 12-h dark. The mice had free access to water and food, and their padding and cages were changed once every two days.

### 2.2 PH mice modeling

All groups of mice were narcotized using 1% sodium pentobarbital (40 mg/kg). Next, the sham group received laparotomy only, and the remaining three model groups were subjected to both laparotomy and subsequent PH. The operation steps are as follows: (1) fix the narcotized mice properly and an incision on the lower edge of the right upper abdominal costal arch; (2) enter the abdominal cavity and mobilize the liver; (3) use a surgical thread to ligate the vessels at the portal area of the left lateral lobe of the liver and use a high-frequency electrocautery to cut off the left lateral lobe; (4) suture the abdomen after ensuring that there was no significant bleeding or damage. After entering the stage of recovery from anesthesia, the mice were placed in an incubator (settings: 37℃; constant temperature) for ventilation till the mice recovered and behaved normally and actively. After the surgery, an EMLA cream mixture (ingredients: 2.5% lidocaine and 2.5% prilocaine) was applied to the wound site at an interval of 8 h for easing pains. Note: all mice were inoculated with the same doses of drugs for anesthesia and analgesic treatment.

### 2.3 Tests in Morris water maze (MWM)

All groups of mice were tested in a MWM on days 1, 3, and 7 after the surgery successively. This MWM system (DMS-2) was manufactured by the Institute of Materia Medica, affiliated with the Chinese Academy of Medical Sciences (Beijing, China). In this system, a circular stainless-steel water tank was provided. This tank was 120 cm in diameter and 50 cm in height. The tank was equally divided into four quadrants. In each quadrant, different colored papers were fixed at the midpoint of the inner wall and above the water surface to serve as visual markers for the mice to locate the platform. A circular platform (9 cm in diameter; 20 cm in height) was positioned at the center point of the target quadrant and 1 cm below the water surface. The entire water tank was surrounded by a blackout curtain to avoid light completely. Before starting the test, the tank was filled with water, which was then mixed with titanium dioxide (a white dye) and stirred till the water became milky white. In the testing process, the incandescent lamp outside the water tank was turned on, with its light directly shining on the curtain and evenly reflecting across the tank. Right above the maze, a camera was mounted and connected to a display system to simultaneously record the mice’s movement paths. In the end, the obtained outcomes were processed using an MWM data collection and analysis system.

Place Navigation Test: The platform was fixed in the target quadrant and remained in the same position throughout the tests. Mice were trained within the same period, four times daily. Every day, the quadrant for mice to enter water for the first time was selected randomly. On the same day, each mouse was trained in a different quadrant, respectively. When entering the same quadrant, the mice entered the water from the same position. Each swimming cycle was 90 s. If a mouse found the preset platform and stayed for more than 3 s on the platform, it was judged that this mouse found the platform successfully. The time a mouse took from entering water to finding the platform was treated as the escape latent period. If a mouse failed to find the platform within 90 s, the mouse would be guided to the platform, staying for 15 s. In this case, the mouse’s escape latent period was recorded as 90 s. This test was conducted on days 1, 3, and 7 post-surgery, respectively, with the escape latent period and total swimming distance recorded within 90 s.

Spatial Search Test: Following the above test, the platform was removed from the target quadrant. The mouse was placed in a randomly chosen quadrant, with the image collection and analysis system automatically recording the search time the mouse stayed in the quadrant where the platform was originally positioned, the search distance, and the frequency of accurate crossings over this quadrant within 90 s.

### 2.4 Collection and preparation of samples

Aged mice were euthanized with the cardiac puncture method on day 3 post-PH, followed by separation of the hippocampal tissue using the following procedures: (1) fix the mice narcotized with 1% sodium pentobarbital (P3761) prepared by Merck Millipore, Billerica (MA, USA); (2) use scissors to cut open the skin and ribs of the thoracic cavity, exposing the heart and liver; (3) insert an injection needle into the left ventricle of the mouse’s heart and cut open the right atrial appendage to allow blood to flow out; (4) perfuse the heart with pre-cooled phosphate-buffered saline on ice for blood replacement till the fluid flowing out of the right atrial appendage was no longer visibly red; (5) cut open the scalp and skull of the mouse in turn, exposing the brain tissue; (6) cut off the medulla oblongata, obtaining the whole brain tissue; (7) have the brain tissue samples for histopathological observation of hippocampal neurons through Nissl staining, TUNEL staining, and immunohistochemical assays placed in 4% paraformaldehyde (P1110) supplied by Solarbio (Beijing, China) and those for western blot and ELISA analyses placed in labeled pre-cooled microcentrifuge tubes without nucleases and stored at −80°C.

### 2.5 Nissl staining for hippocampal tissue

Referring to the research method adopted by Hu et al. [33], we carried out the following procedures for this assay: (1) immerse the hippocampal tissue samples of each experimental mouse (five mice from each group) in 4% paraformaldehyde and culture overnight in a 4°C refrigerator; (2) embed the hippocampal tissue samples in paraffin, then cut the paraffin blocks into slices (thickness: 5 µm) and dewax the slices; (3) dehydrate the slices in a graded series of ethanol, immerse in xylene, and then re-hydrate in gradient concentrations of ethanol; (4) add 1% thionine (G3668) supplied by Solarbio to the slices, followed by 30 min of hydration at 60℃; (5) hydrate the slices immersed in gradient concentrations of ethanol for dehydration, clean in xylene, and then mount with neutral balsam (G8590) sourced from Solarbio; and (6) observe and photograph the hippocampal forms under a light microscope (BX43) provided by Olympus (Tokyo, Japan).

### 2.6 Tunel staining for hippocampal tissue

For this assay, TUNEL assay kits (No.: C1091) were provided by Beyotime Biotechnology (Shanghai, China) and DAPI solution. The apoptotic status of neuronal cells in hippocampal tissue was determined using the TUNEL assay kits as per their user manuals. The detailed steps are as follows: (1) dewax the hippocampal tissue slices, dehydrate the slices using a graded series of ethanol, and clean the slices using xylene; (2) rinse the slices thrice using PBS; (3) add 20 µg/mL proteinase K solution onto the slices, culturing for 30 min at room temperature; (4) rinse the slices with PBS and then put in 3% H_2_O_2_, and incubate for 15 min; (5) rinse the slices with PBS again and administer 50 µL of TUNEL reaction mixture and incubate 60 min in the dark with 37℃ for reaction; (6) rewash the slices using PBS and add DAPI solution for staining, keeping in the dark and at the room temperature for 5 min incubation; (7) use PBS to wash the slices to get rid of DAPI; (8) use an anti-fade mounting medium to seal the remaining matter; and (9) observe five random high-power fields of the seal sheet under a microscope and quantify the TUNEL-positive cells.

### 2.7 Immunofluorescence for hippocampal tissue

The steps of this experiment are as follows: (1) the paraffin sections of the hippocampus were dewaxed and hydrated and placed in a citric acid solution, and microwave treatment was performed to expose the antigen; (2) the sections were dripped with blocking solution to cover the tissue for 30 min; (3) NeuN (ab104224 and IBA-1 (ab178847) antibodies were added and incubated overnight at 4℃; (4) the sections were washed with PBS and incubated with fluorescent secondary antibody (ab150077) for 1 h; and (5) after sealing the film, a microscope was used to observe and take images.

### 2.8 Western blot assay

In this assay, Tris-buffered saline-Tween (TBST), polyvinylidene fluoride membranes (Merck Millipore Corp., Billerica, MA, USA), the anti-NeuN (ab177487; prepared by Abcam, Waltham, MA, USA), anti-p-p65 (ab76302), anti-p65 (ab32536), anti-NLRP3 (ab263899), anti-iNOS (ab3523), anti-CD206 (ab300621), anti-cleaved caspase-1 (ab256469), anti-caspase-1 (ab138483), anti-GSDMD (ab219800), anti-GSDMD-N (ab215203), anti-Bmal1 (ab235577), anti-β-actin (ab8227), anti-*N*-methyl-d-aspartate receptor (NMDAR) subunit 2B (NR2B, ab254356), anti-brain-derived neurotrophic factor (BDNF, ab216443) and horseradish peroxidase-labeled secondary antibody (ab6721) were used. Enhanced chemiluminescence (ECL) substrate (a Pierce ECL western blotting substrate) was provided by Thermo Scientific. The computer picture analysis system ChemiDoc XRS+ was provided by Bio-Rad (Hercules, CA, USA). The assay steps are detailed as follows: (1) extract total proteins from the hippocampal tissues of each mouse group (100 µg from each group) using RIPA lysis buffer; (2) perform SDS-based polyacrylamide gel electrophoresis on the extracted proteins and then transfer the proteins to polyvinylidene fluoride membranes; (3) block the membranes for 2 h at room temperature in 0.1% TBST solution containing 5% bovine serum albumin; (4) add primary antibodies anti-NeuN (diluted to 1:1,000), anti-p-p65 (diluted to 1:5,000), anti-p65 (diluted to 1:1,000), anti-iNOS (diluted to 1:1,000), anti-CD206 (diluted to 1:1,000), anti-NLRP3 (diluted to 1:1,000), anti-Cleaved caspase-1 (diluted to 1:1,000), anti-caspase-1 (diluted to 1:1,000), anti-GSDMD (diluted to 1:1,000), anti-GSDMD-N (diluted to 1:1,000), anti-Bmal1 (diluted to 1:1,000), and anti-β-actin (diluted to 1:1,000) to the solution, incubating overnight at 4°C; (5) wash the two groups of cells for three repetitions using TBST and add horseradish peroxidase-labeled second antibody (diluted to 1:2,000) to the obtained protein strips, incubating at the room temperature for 2 h; (6) dropwise add ECL substrate to the protein strips, wait 1–3 min for the reaction; and (7) use the ChemiDoc XRS+ to measure taking β-actin as an internal reference.

### 2.9 Immunohistochemical assay

In this assay, the citrate antigen retrieval solution (C1032) was provided by Solarbio; the primary antibody anti-Bmal1 (ab230822) and the secondary antibody IgG (ab6721) were supplied by Abcam; diaminobenzidine (DAB, Numbered 24002) was prepared by Thermo Scientific (Waltham, MA, USA); microscope Eclipse 80i was obtained from Nikon (Chiyoda-ku, Tokyo, Japan); and the ImageJ 1.47n software was developed by Wayne Rasband (National Institute of Health, Bethesda, MD, USA). This assay was conducted using a method appropriately modified as per the method reported in the literature [34,35]. The specific steps are as follows: (1) place the hippocampal tissue samples of mice in 4% paraformaldehyde and storing in a refrigerator set at 4°C overnight; (2) dehydrate the samples, embed them in paraffin, and then slice the block into pieces (thickness: 5 µm); (3) perform antigen retrieval using citrate antigen retrieval solution and use 0.01 M PBS (comprising 5% bovine serum albumin and 0.1% Triton X-100) to block the slices for 1 h at the room temperature; (4) incubate the slices overnight at 4 °C using the anti-Bmal1 (diluted to 1:1,000); (5) administer IgG (diluted to 1:2,000) to the slices and incubate for 2 h; (6) add DAB to develop the images of the slices; (7) use the microscope Eclipse 80i to observe and photograph and the staining status on the slices; and (8) analyze the density using the Image J 1.47n software.

### 2.10 Enzyme-linked immunosorbent assay

Hippocampal tissue samples were extracted with the method given in Section 2.4. Then, the samples were tested using the IL-1β ELISA kit (Numbered ab197742) and TNF-α ELISA kit (Numbered ab208348) supplied by Abcam, respectively, in accordance with the user manuals of the kits. Finally, the optical densities of these two inflammatory cytokines were determined at a wavelength of 450 nm, and their expression levels were compared.

### 2.11 Analysis of the obtained data

The obtained data were analyzed statistically using the SPSS 26.0 designed by SPSS Inc., Chicago, IL, USA. All the data were processed using the Kolmogorov–Smirnov method to examine their normal distribution. All the measured data satisfying normal distribution are expressed as the mean ± standard error (i.e., \[\bar{x}]\] ± s). The comparison between two groups of samples was conducted through unpaired *t*-tests, while comparisons among at least three groups were conducted using the one-way ANOVA method. *P < 0.05 indicates a large difference from the control group; ^#^ *P* < 0.05 signifies that there is a marked difference from the NS group.


**Ethical approval:** The research related to animals’ use has been complied with all the relevant national regulations and institutional policies for the care and use of animals. The study was approved by the Ethics Committee of Shaanxi Provincial Cancer Hospital.

## 3 Results

### 3.1 Subanesthetic dose improved the cognitive function of ketamine in PH-aged mice

**Figure 1:**
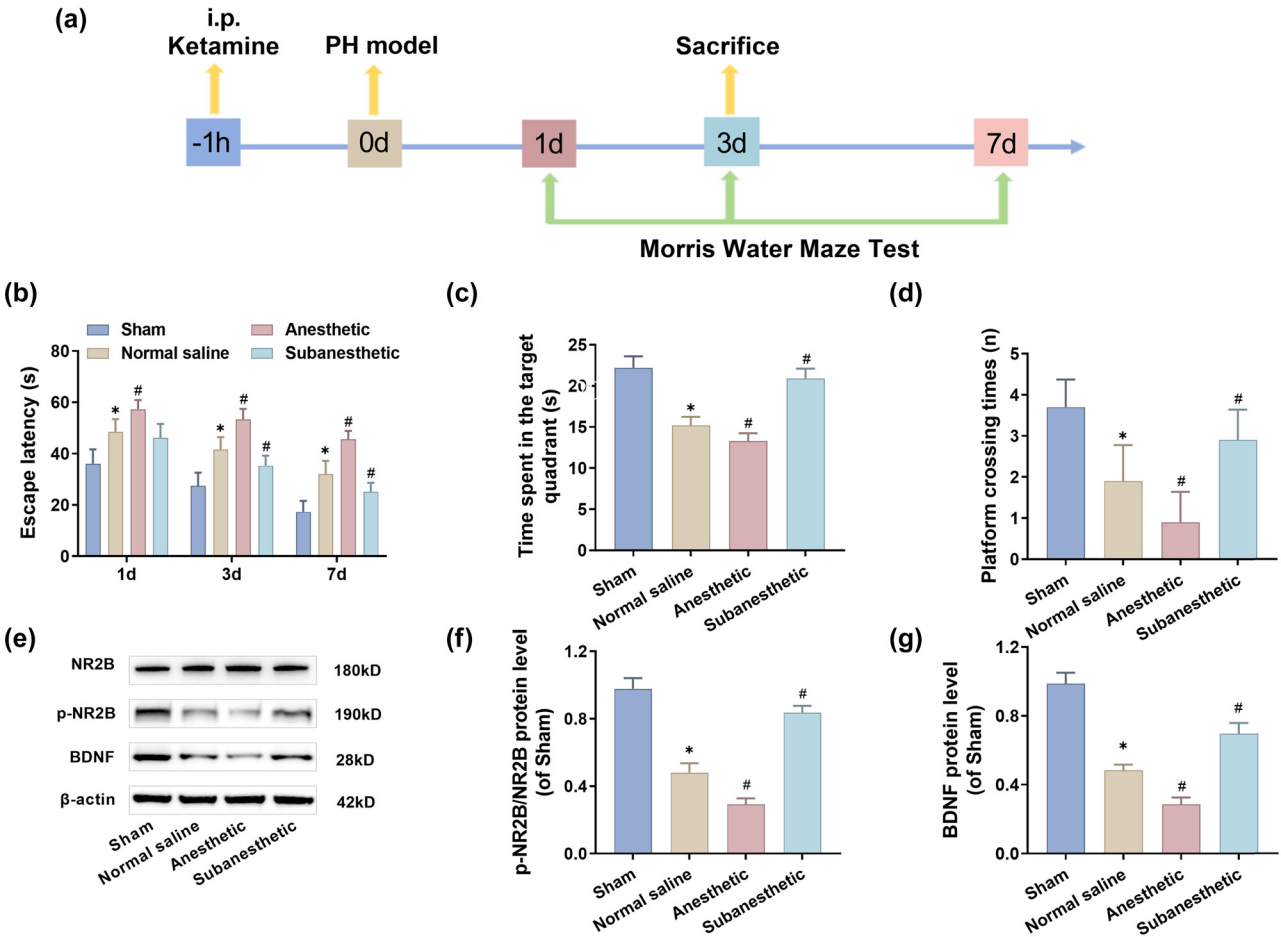
Subanesthetic dose improved the cognitive function of ketamine in PH-aged mice (*signifies a comparison with the sham group; #signifies a comparison with the normal saline group; &signifies a comparison with the subanesthetic group). (a) Timeline design for the test. (b–d): Escape latent period, duration staying in the target quadrant, and frequency of crossings over the platform. (e–g) p-NR2B and BDNF protein levels expressed in the hippocampus as tested by western blotting.

To ascertain how different doses of ketamine affect the memory and learning ability of post-PH-aged mice, we conducted the MWM tests on mice on days 1, 3, and 7 post-surgery, respectively. Figure 1a illustrates the test methods and flowchart of this survey. The behavioral test results reveal the following: First, compared to the sham group, post-PH mice in the other three groups exhibited significantly prolonged escape latent period on days 1, 3, and 7 post-operation (Figure 1b); the frequency of crossings over the platform and the time staying in the target quadrant both showed varying degrees of decline (Figure 1c and d). This outcome indicates that the mice’s memory and learning ability were impaired by PH. Hence, an animal model with cognitive dysfunction was successfully established. Second, in comparison with the NS group, mice in the anesthetic group experienced a longer escape latent period, stayed a shorter time in the target quadrant, and crossed over the platform less frequently, while the mice in the subanesthetic group displayed the opposite behaviors (Figure 1b–d). In addition, this study also found that PH significantly reduced the expression of p-NR2B and BDNF proteins, which further indicated that mice had postoperative cognitive dysfunction. Compared with the NS group, the expression of p-NR2B and BDNF protein in the anesthesia group was significantly decreased, and the expression of p-NR2B and BDNF proteins in the sub-anesthesia group was significantly increased (Figure 1e–g). These phenomena prove that subanesthetic doses of ketamine helped ameliorate the postoperative cognitive functions in aged mice.

### 3.2 Subanesthetic dose of ketamine improved the pathological damage of hippocampal neurons in PH-aged mice

**Figure 2:**
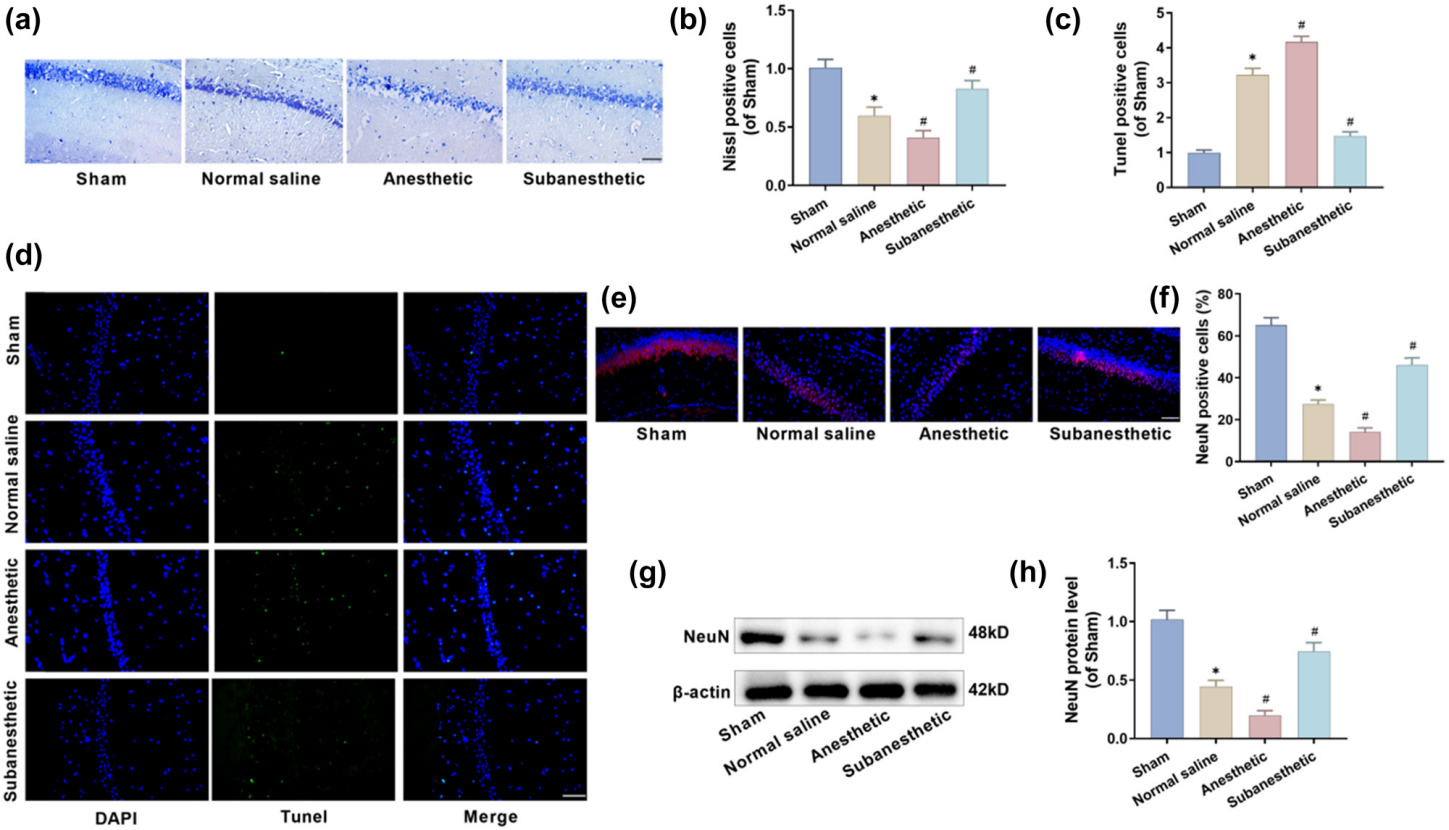
Subanesthetic dose of ketamine improved the pathological damage of hippocampal neurons in PH-aged mice. (a) and (b) Nissl-stained hippocampus (×40, 50 μm). (c) and (d) The hippocampal neuronal apoptotic rate tested by tunnel staining (×40, 50 μm). (e) and (f) The number of NeuN-positive neurons in the hippocampus was detected by immunofluorescence (×40, 50 μm). (g) and (h) NeuN protein levels expressed in the hippocampus as tested by western blotting.

Through Nissl staining, Tunel staining, immunofluorescence, and western blot assays, the pathological damage to hippocampal neurons in mice with cognitive dysfunction was examined. The Nissl staining results show that the sham group had clear and intact neurons with abundant Nissl bodies. In contrast with the sham group, mice in the other three model groups exhibited fewer Nissl-positive cells and lighter staining of Nissl bodies in the hippocampal neurons: mice in the NS group had disordered neurons and shrunken nuclei, along with a substantial loss of Nissl bodies; exacerbated neuronal damage was observed in the anesthetic group in comparison with the NS group; the conditions in the NS group were seen relieved evidently in the subanesthetic group (Figure 2a and b). The Tunel staining results indicate a higher number of Tunel-positive cells in the other three model groups than the sham group, suggesting an increment in the hippocampal neuronal apoptotic rate in aged mice post-PH. Notably, this rate in the subanesthetic group was significantly lower than that in the NS group (Figure 2c and d). Additionally, the results of testing on the neuronal cell marker protein NeuN (Figure 2e–h) also confirmed that there were more NeuN-positive cells in the hippocampus of the sham group, and the cells were multi-layered and closely arranged; the NeuN-positive cells in the hippocampus of the other three model groups were significantly reduced, the structure was loose (Figure 2e and f), and the level of NeuN protein was also significantly reduced (Figure 2g and h). However, the NeuN-positive cells and protein levels in the subanesthetic group were significantly higher than those in the NS group, indicating that, contrary to the normal anesthetic dose, the subanesthetic dose of ketamine can effectively mitigate postoperative neuronal injuries in aged mice.

### 3.3 Subanesthetic dose of ketamine improved hippocampal neuroinflammation in PH-aged mice

**Figure 3:**
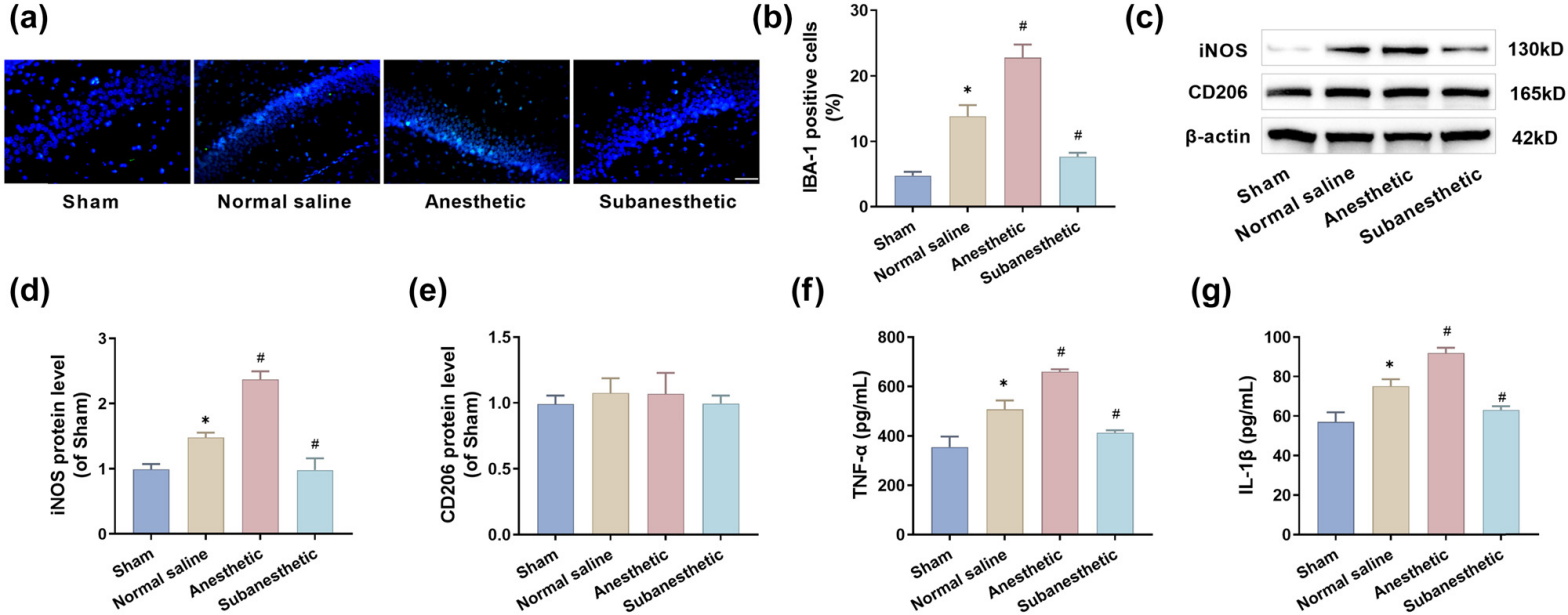
Subanesthetic dose of ketamine improved hippocampal neuroinflammation in PH-aged mice. (a) and (b) The expression of IBA-1 in hippocampus (CA1 region) was detected by immunofluorescence (×40, 50 μm), (c)–(e) Western blot was used to detect the expression of iNOS and CD206 in hippocampus, and (f) and (g) the levels of TNF-α and IL-1β in hippocampus were detected by ELISA.

Neuroinflammation associated with surgical trauma is an important pathogenic factor of postoperative cognitive dysfunction, and microglia play an important role in it. Immunofluorescence results showed that IBA-1-positive cells in the hippocampal CA1 region of PH mice were significantly increased, and the number of IBA-1-positive cells in the anesthesia group was further increased, while the IBA-1-positive cells in the sub-anesthesia group were significantly reduced (Figure 3a and b), indicating that subanesthetic doses of ketamine inhibited microglial activation. Moreover, the M1 polarization marker iNOS in the hippocampus was significantly increased after PH, further increased after the intervention of anesthetic dose of ketamine, and significantly decreased after the intervention of subanesthetic dose of ketamine. However, there was no difference in the M2 polarization marker CD206 among the four groups (Figure 3c–e), indicating that neuroinflammation caused by PH might be related to M1 microglia. Finally, ELISA results also confirmed that a subanesthetic dose of ketamine could alleviate postoperative hippocampal neuroinflammation in elderly mice. The contents of TNF-α and IL-1β increased significantly after PH, but decreased significantly in the subanesthetic group (Figure 3f and g).

### 3.4 Subanesthetic dose of ketamine-mediated neuronal pyroptosis in PH-aged mice by inhibiting the NF-κB/NLRP3 pathway and improving postoperative cognitive function

**Figure 4:**
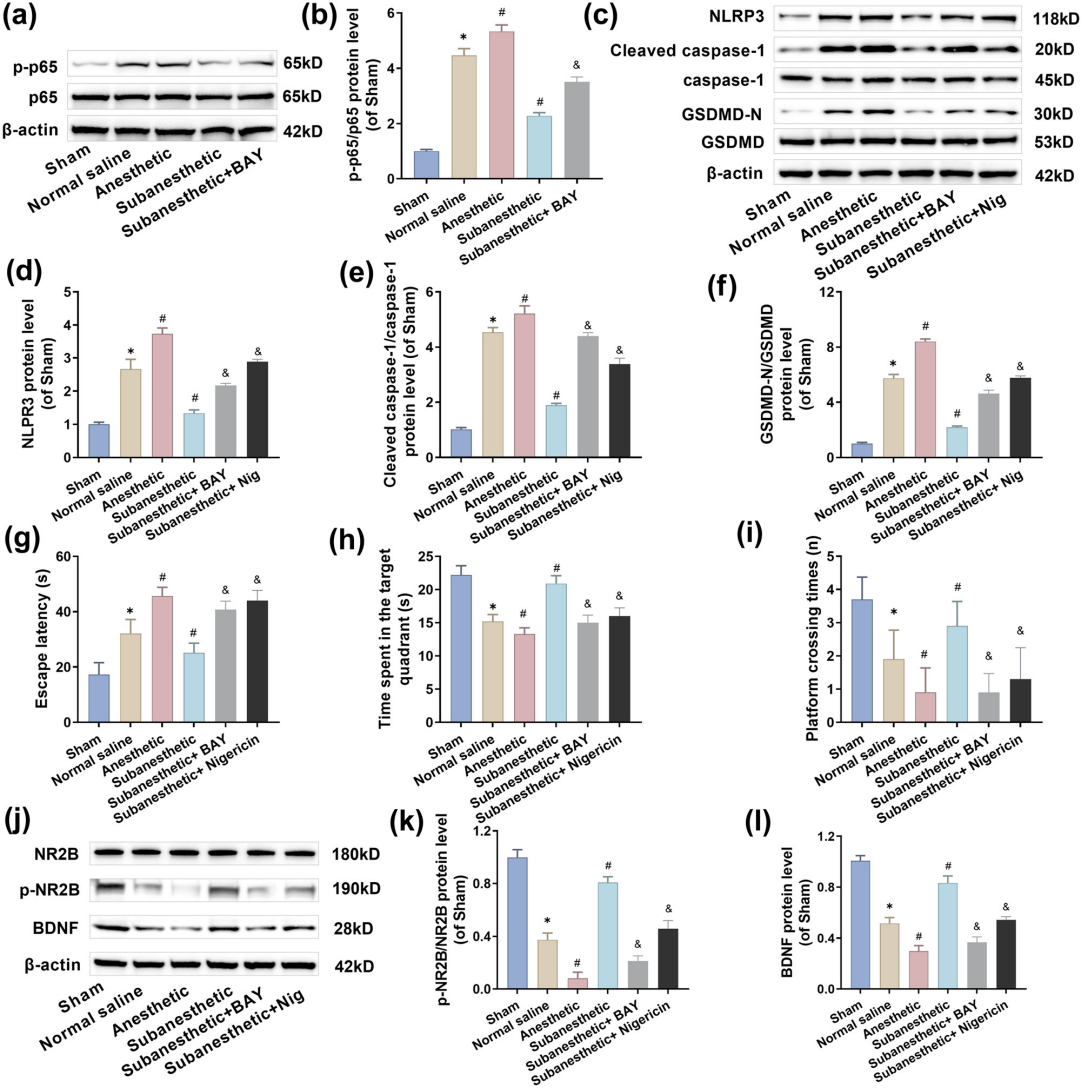
Subanesthetic dose of ketamine mediated neuronal pyroptosis in PH-aged mice by inhibiting NF-κB/NLRP3 pathway and improving postoperative cognitive function. (a)–(f) The p-NF-κB p65, NF-κB p65, NLRP3, cleaved caspase-1, caspase-1, GSDMD, and GSDMD-N levels expressed in the hippocampus, as tested by western blotting. (g)–(i) Escape latent period, duration staying in the target quadrant, and frequency of crossings over the platform in the MWM experiment. (j)–(l) p-NR2B and BDNF protein levels expressed in hippocampus, as tested by western blotting.

Neurocognitive functions are critically regulated by neuroinflammation, as widely acknowledged in scientific research [36]. NLRP3 inflammasome, if activated, may trigger its downstream inflammatory cytokines, leading to neuroinflammation and subsequent pyroptosis of cells. To investigate the involvement of ketamine in post-PH-aged mice’s neuroinflammation, hippocampal tissues were collected from aged mice after ketamine, NF-κB agonist BAY 11-7085, and NLRP3 agonist Nigericin intervention, and the expressed levels of cytokines involved in the NF-κB/NLRP3 pathway were assessed. As revealed through western blotting, compared with the sham group, the hippocampal tissues of mice in the other three model groups encountered upregulated levels of pathway protein p-p65/p65, NLRP3, cleaved caspase-1, and GSDMD-N. Compared with the NS group, the protein level of the anesthesia group was further upregulated, while that of the subanesthesia group was significantly reduced; after the application of BAY 11-7085 and Nigericin on the basis of subanesthesia, these protein levels were significantly increased (Figure 4a–f). Moreover, the MWM experiment showed that compared with the subanesthesia group, the escape latency of mice was significantly increased after the application of BAY 11-7085 and Nigericin, the target quadrant residence time and the number of crossing platforms were significantly reduced (Figure 4g–i), and the levels of p-NR2B and BDNF proteins were also significantly reduced (Figure 4j–l). It is suggested that a subanesthetic dose of ketamine inhibited the NF-κB/NLRP3 signaling pathway and inhibited neuronal pyroptosis, which explains why a subanesthetic dose of ketamine can improve postoperative cognitive function in aged mice.

### 3.5 Subanesthetic dose of ketamine upregulated the expression of Bmal1 in the hippocampus of PH-aged mice

**Figure 5:**
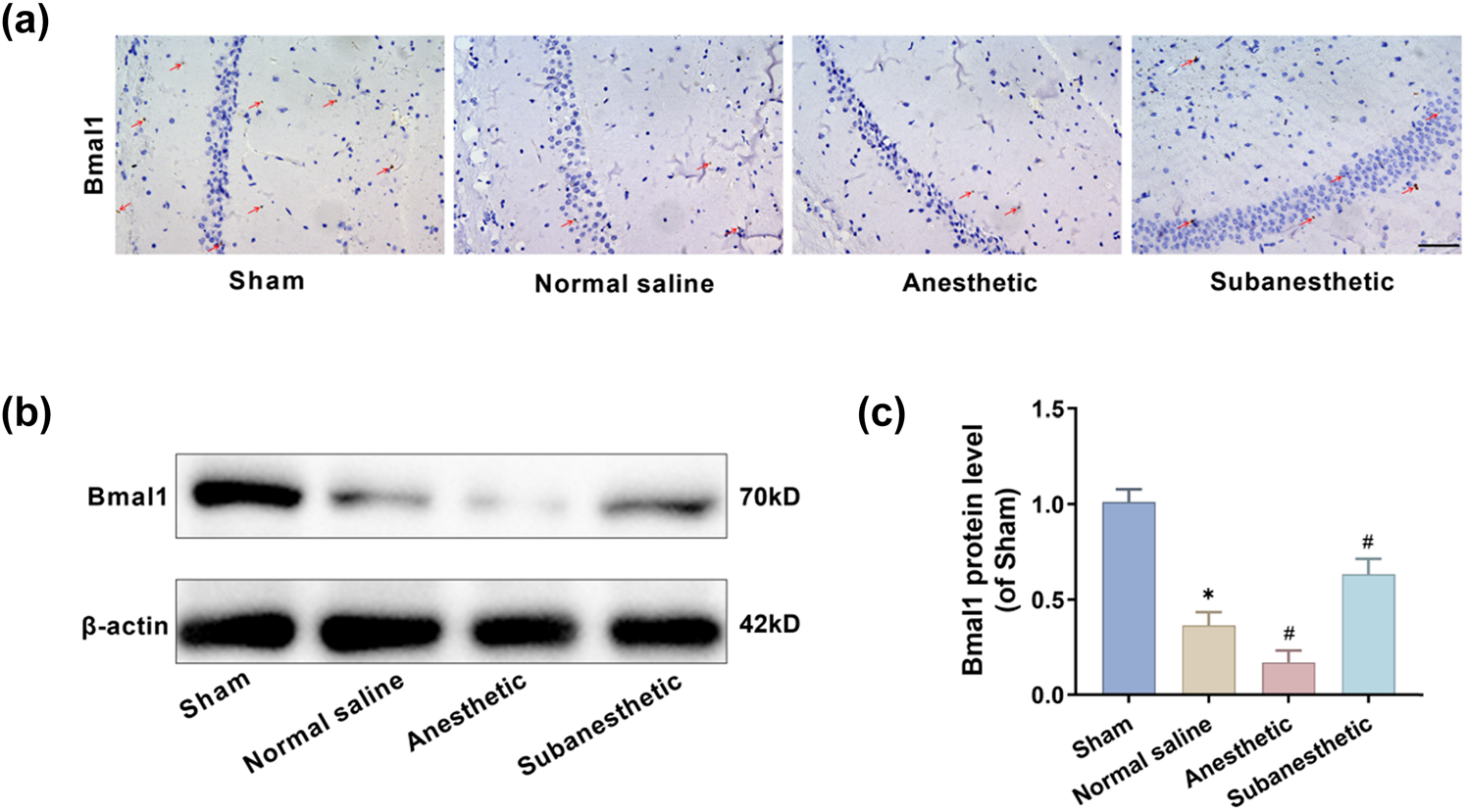
Subanesthetic dose of ketamine upregulated the expression of Bmal1 in the hippocampus of PH-aged mice. (a) The expressed level of Bmal1 in the hippocampus as tested immunohistochemically (×40, 50 μm). (b) and (c) The expressed level of Bmal1 in the hippocampus, as tested by western blotting.

Immunohistochemical and western blot assays reveal that the Bmal1 gene in aged mice post-PH was downregulated. Regarding the number of Bmal1-positive cells, the three model groups ranked as follows: subanesthetic group > NS group > anesthetic group (Figure 5a); a similar trend was exhibited in the levels of Bmal1 protein (Figure 5b and c). These outcomes match the findings from the author’s previous study [32]. Above all, subanesthetic doses of ketamine appeared to upregulate the level of the Bmal1 gene and suppress the NF-κB/NLRP3 signaling pathway. This action contributed to the recovery of the postoperative cognitive functions in aged mice. Hence, it was hypothesized that there was a reciprocal regulatory relationship between Bmal1 and NF-κB/NLRP3, and subanesthetic doses of ketamine might inhibit hippocampal neuronal pyroptosis by modulating the NF-κB/NLRP3 signaling pathway via the Bmal1 gene.

### 3.6 Subanesthetic dose of ketamine attenuated cognitive impairment and hippocampal neuronal pathological damage induced by Bmal1 knockout in PH-aged mice

**Figure 6:**
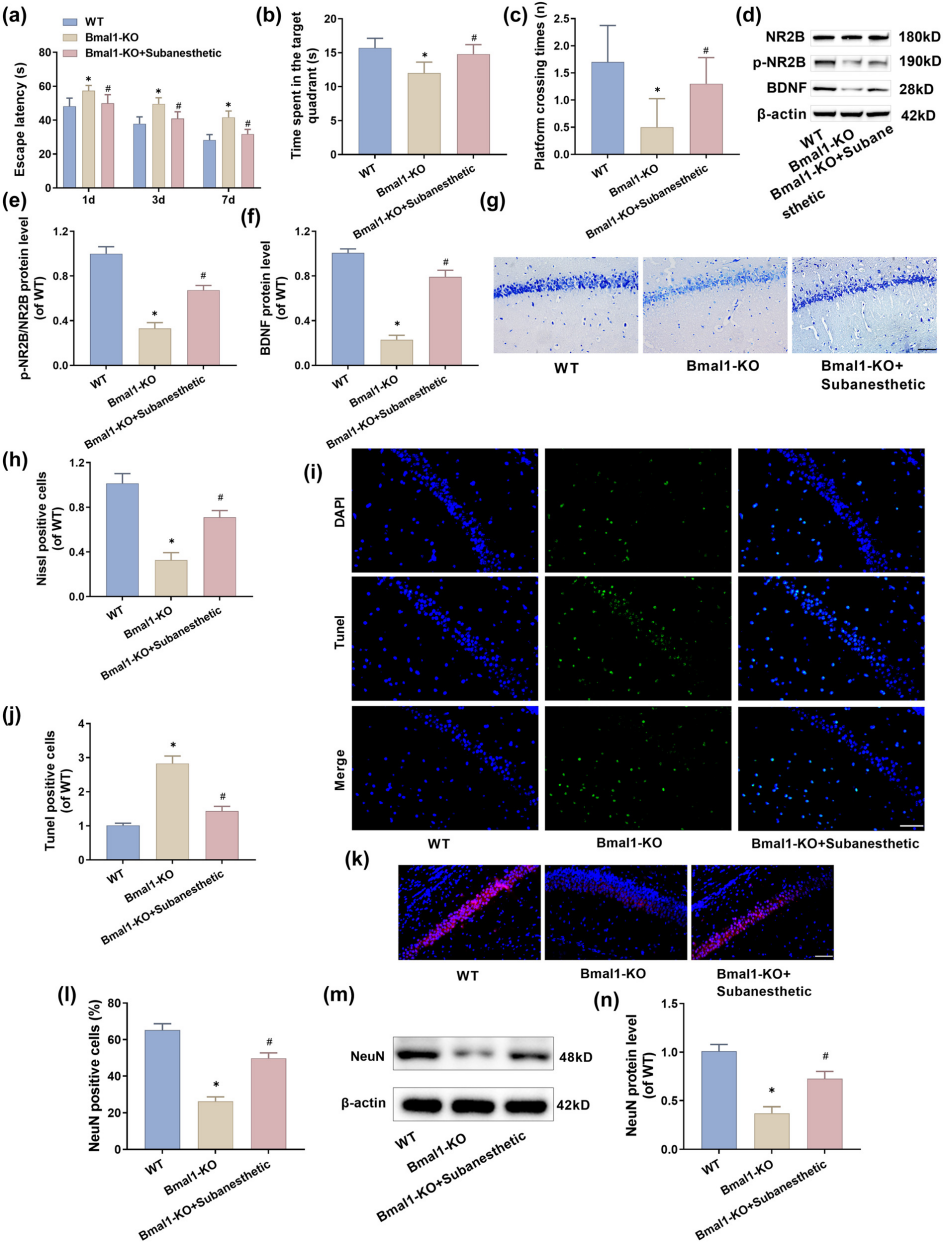
Subanesthetic dose of ketamine attenuated cognitive impairment and hippocampal neuronal pathological damage induced by Bmal1 knockout in aged PH mice (*signifies a comparison with the WT group; #signifies a comparison with the Bmal1-KO group). (a)–(c) Escape latent period, duration staying in the target quadrant, and frequency of crossings over the platform. (d)–(f) p-NR2B and BDNF protein levels expressed in hippocampus, as tested by western blotting. (g) and (h) Nissl-stained hippocampus (×40, 50 μm). (i) and (j) The hippocampal neuronal apoptotic rate tested by tunnel staining (×40, 50 μm). (k) and (l) The number of NeuN positive neurons in the hippocampus was detected by immunofluorescence (×40, 50 μm). (m) and (n) NeuN protein levels expressed in the hippocampus, as tested by western blotting.

To verify Bmal1’s close connection to postoperative cognitive dysfunction in aged mice, subsequent experiments were conducted on aged mice with a congenitally inactive Bmal1 gene. Similarly, such mice were also tested in the MWM on days 1, 3, and 7 after surgery. The results are as follows: First, compared with the WT group, aged mice in the Bmal1-KO group on these 3 days post-operation presented significantly longer escape latent periods (Figure 6a), shortened duration staying in the target quadrant (Figure 6b), and reduced frequency of crossings over the platform (Figure 6c). In addition, p-NR2B and BDNF protein levels were also significantly reduced (Figure 6d–f). This phenomenon indicates that the mice’s memory and learning ability were impaired with the inactivation of the Bmal1 gene. When the aged mice with inactive Bmal1 gene were inoculated with subanesthetic doses of ketamine, some of their cognitive functions were recovered (Figure 6a–f). Thereby, it is verified that Bmal1 is a crucial target by which subanesthetic doses of ketamine repair the postoperative cognitive dysfunction of aged patients. As proved above, subanesthetic doses of ketamine can effectively mitigate neuronal injuries in aged mice post-surgery. By knocking the Bmal1 gene out, how subanesthetic doses of ketamine acted in repairing pathological injuries of hippocampal neurons in post-PH-aged mice was further validated. The results are as follows: First, compared to the WT group, the Bmal1-KO group of mice manifested reduced Nissl-positive cells in their hippocampal neurons (Figure 6g and h), increased Tunel-positive cells (Figure 6i and j), and lowered NeuN-positive cells (Figure 6k and l) and protein levels (Figure 6m and n), indicating an elevated rate of neuronal apoptosis in hippocampal neurons of post-PH-aged mice with Bmal1 gene deactivated. Second, mice in the Bmal1-KO + subanesthetic group showed clearly weakened neuronal apoptosis in hippocampal neurons (Figure 6g–n). These experimental results demonstrate that subanesthetic doses of ketamine can alleviate hippocampal neuronal injuries through neuronal apoptosis, diminishing by elevating the level of the Bmal1 gene.

### 3.7 Subanesthetic dose of ketamine attenuates neuroinflammation and neuronal pyroptosis in the hippocampus of Bmal1 knockout-induced PH-aged mice

**Figure 7:**
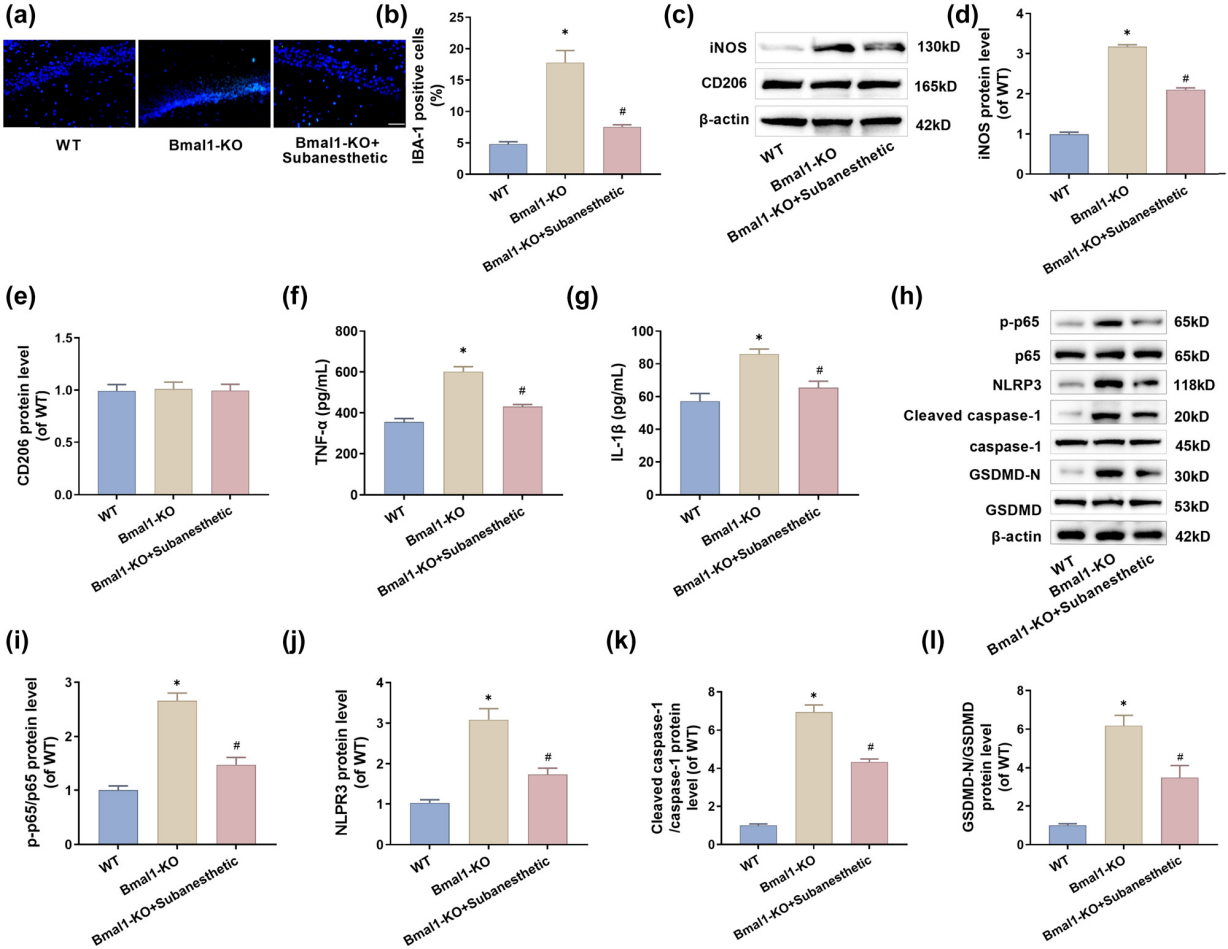
Subanesthetic doses of ketamine ease the NF-κB/NLRP3 pathway-mediated post-PH neuronal pyroptosis in aged mice due to Bmal1 knockout. (a) and (b) The expression of IBA-1 in hippocampus (CA1 region) was detected by immunofluorescence (×40, 50 μm). (c)–(e) Western blotting was used to detect the expression of iNOS and CD206 in the hippocampus. (f) and (g) The levels of TNF-α and IL-1β in the hippocampus were detected by ELISA. (h–l) The p-NF-κB p65, NF-κB p65, NLRP3, cleaved caspase-1, caspase-1, GSDMD, and GSDMD-N levels expressed in the hippocampus, as tested by western blotting.

For validating the previous hypothesis regarding the regulatory relationship between Bmal1 and NF-κB/NLRP3, the expressed levels of cytokines related to the NF-κB/NLRP3 pathway were examined following the deactivation of the Bmal1 gene. Consequently, the following outcomes were obtained: First, in the Bmal1-KO group, the levels of IBA-1-positive cells (Figure 7a and b), M1 polarization marker iNOS (Figure 7c and d) and inflammatory cytokines TNF-α and IL-1β (Figure 7f and g) in the hippocampal region were significantly increased, but there was no significant difference in the level of M2 polarization marker CD206 (Figure 7c and e). Second, the protein expression levels of p-p65, NLRP3, cleaved caspase-1, and GSDMD-N were increased (Figure 7h–l). However, in the Bmal1-KO + subanesthesia group, there was still no significant difference in CD206 levels, and the levels of the remaining indicators were effectively restored (Figure 7a–l). In summary, the Bmal1 gene is suppressive for the NF-κB/NLRP3 pathway. This confirms that subanesthetic doses of ketamine can restrain the NF-κB/NLRP3 signaling pathway by elevating the volume of the Bmal1 gene, and this restraining further leads to alleviation of hippocampal neuronal pyroptosis, thus improving the aged mice’s cognitive dysfunction post-surgery.

## 4 Discussion

As clinically observed in the past, anesthetic doses of ketamine induced postoperative cognitive dysfunction in elderly patients, while subanesthetic doses of ketamine improved this postoperative condition [10]. Against this background, the study was designed and surveyed. As found in the survey, the Bmal1 gene was downregulated, and the release of inflammatory cytokines IL-1β and TNF-α in the hippocampal tissue was enhanced in mice injected with anesthetic doses of ketamine; this condition exacerbated the impairment of postoperative cognitive functions. However, in mice with subanesthetic doses of ketamine, this condition was regulated in opposite trends, improving the postoperative cognitive functions of aged mice post-surgery. Based on these findings, it was speculated that different doses of ketamine might affect postoperative cognitive functions through the clock gene Bmal1. To confirm this speculation, the behaviors of mice were tested and observed with an inactivated Bmal1 gene in an MWM, along with assessing the influences of this gene on the postoperative cognitive functions of such mice. The outcomes prove that the mice with an inactivated Bmal1 gene and injected with subanesthetic doses of ketamine experienced a shorter escape latent period, stayed a longer time in the target quadrant, and frequently crossed over the platform. This phenomenon demonstrates that Bmal1 is a key node in the path subanesthetic doses of ketamine ameliorate postoperative cognitive functions in aged mice. The defect of learning and memory ability is related to the cholinergic neurotransmitter system in the hippocampus. Acetylcholinesterase (AChE) participates in the learning and memory process through the septum–hippocampus–marginal lobe. The activity of AChE directly reflects the synthesis and metabolism of acetylcholine. Inhibition of AChE activity can improve the learning and memory ability of mice in the MWM experiment. Amyloid β-protein (Aβ) is a pathological indicator of postoperative cognitive dysfunction. When Aβ production increases, Aβ_1-42_ accelerates deposition and produces neurotoxicity. Aβ_1-42_ stimulates the phagocytosis of glial cells, leads to the release of inflammatory factors, increases the inflammatory damage of brain tissue, and induces neuronal apoptosis. Inhalation of anesthetics can lead to Aβ oligomerization, resulting in hyperphosphorylation of tau protein, affecting normal axonal transport, leading to synaptic loss and neuronal function damage, eventually leading to neurodegenerative diseases [37–42].

There is a potential relationship between the expression of the NR2B gene and cognitive function. NR2B is mostly distributed in the hippocampus, prefrontal lobe, and related cerebral cortex, which can affect the cognitive function. Zhu et al. [43] found that the cognitive deficits caused by the chronic hypobaric hypoxia rat model were related to the decrease in NR2B gene expression. In the AD model rats with long-term memory deficits, the transcription level of the NR2B gene was increased [44]. BDNF plays an important role in the regulation of neuronal growth and development, the construction of functional neuronal networks, neuroprotective effects, short-term and long-term synaptic interactions that are crucial for cognitive and memory functions, the formation of synaptic structures, and the dynamic regulation of synaptic plasticity [45]. In mental disorders such as depression, BDNF levels are also considered to be an important biomarker [46]. Studies have found that anesthesia/surgery can inhibit the BDNF/TrkB signaling pathway and cause learning disorders. Activation of the BDNF pathway can effectively prevent postoperative cognitive dysfunction in elderly mice [47,48]. In this study, the expression of p-NR2B and BDNF proteins in the NS group was significantly decreased, which also indicated that PH caused postoperative cognitive dysfunction. Compared with the NS group, the expression of p-NR2B and BDNF proteins in the anesthesia group was significantly decreased, and the expression of p-NR2B and BDNF proteins in the sub-anesthesia group was significantly increased, indicating that subanesthetic dose ketamine can improve postoperative cognitive dysfunction.

This study is based on the mechanism study of the PH elderly mice. Subanesthetic dose of ketamine can upregulate the expression of Bmal1, improve the cognitive function injury, and hippocampal neuronal pathological injury in PH elderly mice. Studies have shown that low-dose ketamine can improve cognitive dysfunction after heart, gastrointestinal tract, laparotomy, and other operations in clinical practice [49,50]. Based on the results of this study, we reasonably speculate that the use of subanesthetic dose ketamine may also have an improvement significance in the cognitive function injury after clinical PH, which provides a certain theoretical guidance for the expansion of its application scope. Future studies should focus on exploring whether subanesthetic doses of ketamine play a role in postoperative cognitive dysfunction by up-regulating Bmal1 expression in other types of surgeries.

Similarly, aged mice with the Bmal1 gene active/inactivated were selected and treated with Nissl staining, Tunel staining, and NeuN expression methods. The purpose was to analyze the actions of subanesthetic doses of ketamine in counteracting neuronal pathological injury in the hippocampal tissue of aged mice. Located in the cell nuclei and perinuclear cytoplasm of most neurons in the CNS of mammalian species [51], the neuronal nuclear protein NeuN has been extensively applied to immunohistochemical research on neuronal differentiation. This protein can be treated as a neuronal-specific marker for assessing the functions of neurons under normal/pathological conditions [52–55]. As discovered from the assays, the group treated with subanesthetic doses of ketamine exhibited a larger number of Nissl-positive neuronal cells, a lower rate of neuronal apoptosis, and a higher level of NeuN, hence alleviating the neuronal injuries of aged mice post-surgery. Because neuronal injury affects cognitive abilities, leading to cognitive dysfunction in mice, the above assay outcomes reconfirm that Bmal1 is significant in the subanesthetic doses of ketamine and improves postoperative cognitive functions in aged mice.

The hippocampus is an important part of the brain. A large number of studies have confirmed that the hippocampal CA1 region plays an important role in improving postoperative cognitive dysfunction. Esketamine has a protective effect on neuronal apoptosis and reduces neuroinflammation, thereby reducing postoperative cognitive dysfunction in elderly mice [56]. Ketamine can alleviate neuronal degeneration and microglia activation in the hippocampal CA1 region after surgery and improve cognitive dysfunction caused by anesthesia and surgery [13]. It has been reported that neuroinflammation is a key factor in postoperative cognitive dysfunction. The inflammatory response in the brain usually involves a large number of neuronal necrosis or death and microglia activation [57]. Microglia are resident immune cells in the CNS. When activated by external stimuli, they can highly express IBA-1 and secrete a large number of cytokines [58]. According to the different secretion of cytokines, microglia are divided into M1 type (marker iNOS) and M2 type (marker CD206). M1-type microglia are characterized by increased release of pro-inflammatory factors such as IL-1β, TNF-α, and IL-6, triggering neuroinflammatory responses. In contrast, M2 microglia upregulate the expression of anti-inflammatory mediators and neurotrophic factors, such as IL-10, to play a neuroprotective role and promote the repair of nerve injury [59,60]. This experiment found that a subanesthetic dose of ketamine can significantly reduce the increase of IBA-1, iNOS, TNF-α, and IL-1β levels caused by PH, indicating that a subanesthetic dose of ketamine can reduce neuroinflammation by reducing microglia activation. In addition, the hippocampal CA3 region has also been confirmed to be closely related to the occurrence of postoperative cognitive dysfunction. Increased apoptosis, decreased synapses, and reduced plasticity can lead to postoperative cognitive dysfunction [61]. The development of the hippocampal dentate gyrus is also closely related to the cognitive function of the body, and neural stem cells in the dentate gyrus play an important role in it. In this study, we focused on the effects of the hippocampal CA1 region on postoperative cognitive dysfunction in aged mice, and have not studied the effects of other hippocampal regions, such as CA3 and dentate gyrus, on postoperative cognitive dysfunction. In the future, this study will explore the nerve injury and inflammation in different hippocampal regions to further enrich the relevant basis of ketamine’s neuroprotective effect to improve postoperative cognitive dysfunction in elderly mice.

When the body performs PH and other traumatic operations, it can cause a stress response, promote the body to release a large number of inflammatory factors, trigger peripheral inflammatory response, and then activate glial cells in the CNS and release a large number of inflammatory factors, damage neurons, and lead to cognitive dysfunction [62–64]. The MWM experiment showed that the escape latency of mice after PH was significantly prolonged, and the frequency of crossing the platform and the time of staying in the target quadrant were reduced, indicating that PH surgery impaired the memory and learning ability of mice and caused postoperative cognitive dysfunction. In the subsequent histopathological and molecular studies, the number of IBA-1 positive microglia in the hippocampus of mice after PH was significantly increased, and the expression of TNF-α and IL-1β was increased. Subsequently, Nissl staining showed that the neurons after PH were disordered, a large number of Nissl bodies were missing, a large number of neurons in the hippocampus were apoptotic, and the expression of NeuN was decreased, which indicated that neuroinflammation and injury occurred in the hippocampus of mice, leading to cognitive dysfunction. After the intervention of subanesthetic dose of ketamine, the escape latency of mice decreased, the frequency of crossing the platform and the time of staying in the target quadrant increased, and the number of IBA-1 positive microglia, the number of Nissl bodies, the expression of NeuN, TNF-α, and IL-1β in hippocampus increased, indicating that subanesthetic dose of ketamine can reduce neuroinflammation and injury, thereby improving the cognitive dysfunction of PH elderly mice.

Furthermore, the association of neuronal pyroptosis with postoperative cognitive dysfunction in mice was probed. Pyroptosis is a type of programmed cell death relying on caspase-1 and initiated by inflammasomes [65]. The NF-κB/NLRP3/caspase-1 signaling pathway acts crucially in the process of pyroptosis, where NF-κB induces the expression of NLRP3 to activate inflammasomes [20,35,66,67]. P65, a subunit of the NF-kB family, can activate the NF-kB pathway, raising the transcriptional activity of this pathway, thereby modulating multiple signaling pathways and inflammatory responses in cells; p-p65, a phosphorylated form of p65, can impact the transcriptional activity of p65 [68,69]. The activation status of NF-κB can be verified by measuring the expressed levels of p-p65/p65. The NF-κB once activated can further activate NLRP3, inducing the cleavage of caspase-1 into its active form (i.e., cleaved caspase-1), leading to the cleavage of GSDMD into the pore-forming GSDMD-N terminus, ultimately causing cell rupture [33,70–74]. In the meantime, this process drives the generation of such pro-inflammatory cytokines as IL-1β and IL-18, triggering neuronal pyroptosis and inflammatory responses [75–81]. As already reported in the literature, blocking the NLRP3/caspase-1 axis helps to alleviate ketamine-triggered hippocampal neuronal pyroptosis and cognitive dysfunction in newborn rats [74]. The NF-κB/NLRP3 pathway can mediate neuronal pyroptosis in mice and improve postoperative cognitive dysfunction [82]. In addition, studies have found that Bmal1 is closely related to NF-κB. Knockout of Bmal1 can activate the NF-κB pathway and the expression of downstream inflammatory factors, exacerbating the severity of periodontitis in mice [83]. Bmal1 can reduce the release of inflammatory factors IL-18 and IL-1β by inhibiting the NF-κB/MMP9 pathway in mice [84]. Bmal1 regulates *propionibacterium acnes*-induced inflammation through the NF-κB/NLRP3 axis [85]. Therefore, this study investigated whether ketamine can play a neuroprotective role through the NF-κB/NLRP3/caspase-1 pathway.

As found in this survey, the expressed level of Bmal1 gene was inhibited by anesthetic doses of ketamine; the NF-κB/NLRP3 signaling pathway was stimulated by anesthetic doses of ketamine; the pyroptotic rate in hippocampal neurons of aged mice increased with the injection of anesthetic doses of ketamine; moreover, anesthetic doses of ketamine contributed to the rise in the expressed levels of NLRP3, GSDMD-N, and cleaved caspase-1, increased the escape latency of mice in the MWM experiment, reduced the target quadrant residence time and the number of crossing platforms, reduced the expression of p-NR2B and BDNF proteins, and further impaired the cognitive functions. In contrast, subanesthetic doses of ketamine upregulated the Bmal1 gene, restraining the NF-κB/NLRP3 signaling pathway, postoperative pyroptosis, the expressions of NLRP3, GSDMD-N, and cleaved caspase-1, and the escape latency of mice, and increased the expression of p-NR2B and BDNF proteins, thereby improving the postoperative cognitive functions. In addition, the protein levels of p-NF-κB p65, NLRP3, cleaved caspase-1, and GSDMD-N were significantly increased after the application of NF-κB and NLRP3 agonists on the basis of subanesthetic dose ketamine. The above results demonstrate that the Bmal1 gene can affect hippocampal neuronal pyroptosis by modulating the NF-κB/NLRP3/caspase-1 signaling pathway, while subanesthetic doses of ketamine can elevate aged mice’s cognitive functions by controlling hippocampal neuronal pyroptosis via the Bmal1 gene. We focused on elucidating the mechanism by which ketamine affects postoperative cognitive dysfunction in elderly mice through the NF-κB/NLRP3/caspase-1 pathway, without an extensive in-depth study of other mechanisms. There are many signaling pathways to improve postoperative cognitive dysfunction by exerting neuroprotective effects. Therefore, this study will continue to explore the mechanism by which ketamine improves postoperative cognitive dysfunction, in order to fully reveal the multidimensional effects of ketamine on cognitive function.

In summary, our study shows that a subanesthetic dose of ketamine inhibits NF-κB/NLRP3 pathway-mediated pyroptosis by upregulating Bmal1 expression and improves postoperative cognitive dysfunction in aged mice. However, it is worth noting that only female C57BL/6 mice were used in this study, and the possible effect of gender on the experimental results was not explored. Considering that gender differences may produce different responses in physiological and pathological processes, future studies should include gender factors for grouping to more comprehensively evaluate the therapeutic effect of ketamine on postoperative cognitive dysfunction and the universality of its mechanism. In addition, since ketamine is dose-dependent and long-term use of ketamine has a risk of abuse [86]. However, our study did not explore the effects of long-term use of ketamine and different subanesthetic doses of ketamine on cognitive dysfunction in aged mice. It will be useful for future research, including multiple time points and subanesthetic doses, to further study the effects of ketamine on cognitive dysfunction in aged mice.

## 5 Conclusion

Before and after knocking the Bmal1 gene out, this study examined the levels of NF-κB/NLRP3/caspase-1 pathway-related proteins as well as inflammatory cytokines IL-1β and TNF-α in the hippocampal neuronal cells of aged mice treated with subanesthetic doses of ketamine. The results clarify that subanesthetic doses of ketamine can control neuronal pyroptosis and neuroinflammation by modulating the downstream NF-κB/NLRP3/caspase-1 signaling pathway through the Bmal1 gene, thereby enhancing the cognitive functions post-surgery. This study provides new ideas for unearthing the relation between clock genes and inflammation in the nervous system. This study suggests that Bmal1 may be a new target for improving postoperative cognitive dysfunction in elderly patients. For the advantages of ketamine in animal experiments, there is currently no evidence to support clinical research. It is also necessary to carry out multi-center, large-sample, long-term observation of clinical studies to clarify the safety and efficacy of ketamine. This study will also combine humanized mice and clinical experiments to further verify the conclusions of this study and provide a theoretical basis for clinical treatment to improve postoperative cognitive function. In addition, there may be potential interactions between ketamine and other postoperative drugs or interventions, so it needs to be carefully considered in clinical applications.
